# Use of Pfizer-BioNTech COVID-19 Vaccine in Persons Aged ≥16 Years: Recommendations of the Advisory Committee on Immunization Practices — United States, September 2021

**DOI:** 10.15585/mmwr.mm7038e2

**Published:** 2021-09-24

**Authors:** Kathleen Dooling, Julia W. Gargano, Danielle Moulia, Megan Wallace, Hannah G. Rosenblum, Amy E. Blain, Stephen C. Hadler, Ian D. Plumb, Heidi Moline, Jack Gerstein, Jennifer P. Collins, Monica Godfrey, Doug Campos-Outcalt, Rebecca L. Morgan, Oliver Brooks, H. Keipp Talbot, Grace M. Lee, Matthew F. Daley, Sara E. Oliver

**Affiliations:** ^1^CDC COVID-19 Response Team; ^2^Epidemic Intelligence Service, CDC; ^3^University of Arizona, College of Medicine, Phoenix, Arizona; ^4^Department of Health Research Methods, Evidence and Impact, McMaster University, Hamilton, Ontario; ^5^Watts Healthcare Corporation, Los Angeles, California; ^6^Vanderbilt University School of Medicine, Nashville, Tennessee; ^7^Stanford University School of Medicine, Stanford, California; ^8^Institute for Health Research, Kaiser Permanente Colorado, Denver, Colorado.

The Pfizer-BioNTech COVID-19 vaccine (BNT162b2) is a lipid nanoparticle-formulated, nucleoside mRNA vaccine encoding the prefusion spike glycoprotein of SARS-CoV-2, the virus that causes COVID-19. Vaccination with the Pfizer-BioNTech COVID-19 vaccine consists of 2 intramuscular doses (30 *μ*g, 0.3 mL each) administered 3 weeks apart. In December 2020, the vaccine was granted Emergency Use Authorization (EUA) by the Food and Drug Administration (FDA) as well as an interim recommendation for use among persons aged ≥16 years by the Advisory Committee on Immunization Practices (ACIP) (*1*). In May 2021, the EUA and interim ACIP recommendations for Pfizer-BioNTech COVID-19 vaccine were extended to adolescents aged 12–15 years (*2*). During December 14, 2020–September 1, 2021, approximately 211 million doses of Pfizer-BioNTech COVID-19 vaccine were administered in the United States.* On August 23, 2021, FDA approved a Biologics License Application for use of the Pfizer-BioNTech COVID-19 vaccine, Comirnaty (Pfizer, Inc.), in persons aged ≥16 years (*3*). The ACIP COVID-19 Vaccines Work Group’s conclusions regarding the evidence for the Pfizer-BioNTech COVID-19 vaccine were presented to ACIP at a public meeting on August 30, 2021. To guide its deliberations regarding the Pfizer-BioNTech COVID-19 vaccine, ACIP used the Evidence to Recommendation (EtR) Framework,^†^ and incorporated a Grading of Recommendations, Assessment, Development and Evaluation (GRADE) approach.^§^ In addition to initial clinical trial data, ACIP considered new information gathered in the 8 months since issuance of the interim recommendation for Pfizer-BioNTech COVID-19 vaccine, including additional follow-up time in the clinical trial, real-world vaccine effectiveness studies, and postauthorization vaccine safety monitoring. The additional information increased certainty that benefits from prevention of asymptomatic infection, COVID-19, and associated hospitalization and death outweighs vaccine-associated risks. On August 30, 2021, ACIP issued a recommendation^¶^ for use of the Pfizer-BioNTech COVID-19 vaccine in persons aged ≥16 years for the prevention of COVID-19.

During June 2020–August 2021, ACIP has convened 18 public meetings to review data on the epidemiology of COVID-19 and considerations for use of COVID-19 vaccines, including the Pfizer-BioNTech COVID-19 vaccine (*4*). The ACIP COVID-19 Vaccines Work Group, comprising experts in infectious diseases, vaccinology, vaccine safety, public health, and ethics, has held one to two meetings each week to review COVID-19 surveillance data; evidence for vaccine efficacy, effectiveness, and safety; and implementation considerations for COVID-19 vaccines. After a systematic review of published and unpublished scientific evidence for benefits and harms, the Work Group used a GRADE approach to assess the certainty of evidence for outcomes related to the vaccine, rated on a scale of 1 (high certainty) to 4 (very low certainty). Within the EtR Framework, ACIP considered the importance of COVID-19 as a public health problem, benefits and harms (including GRADE-assessed evidence), patients’ values and preferences, issues of resource use, acceptability to stakeholders, feasibility of implementation, and anticipated impact on health equity. Work Group conclusions regarding the evidence for the Pfizer-BioNTech COVID-19 vaccine were presented to ACIP at a public meeting on August 30, 2021.**

The body of evidence for the Pfizer-BioNTech COVID-19 vaccine was guided by one large randomized, double-blind, placebo-controlled phase II/III clinical trial (*5*) and one small phase I clinical trial (*6*), 26 observational vaccine effectiveness studies, and two postauthorization vaccine safety monitoring systems: 1) the Vaccine Adverse Events Reporting System (VAERS) and 2) the Vaccine Safety Datalink (VSD). VAERS, the national vaccine safety monitoring system managed by CDC and FDA, is based on passive reporting and covers the entire U.S. population. VSD covers nine participating integrated health care organizations serving approximately 12 million persons and identifies possible adverse events after vaccination as well as age, race, ethnicity, and other demographic information from electronic medical records. Updated findings from the ongoing phase II/III clinical trial were based on data from 44,165 enrolled participants contributing approximately 12,000 person-years, with more than one half of participants followed for ≥6 months (August 24, 2020–March 13, 2021). Pooled effectiveness estimates were calculated when multiple observational studies reported data on an outcome, the study periods for which ranged from 3 to 7 months (median = 5 months).

The estimated efficacy of the Pfizer-BioNTech COVID-19 vaccine in the phase II/III clinical trial was based on outcomes that occurred ≥7 days after receipt of the second dose. The demographic characteristics of participants, including age and race, published in December 2020 (*5*), have not changed substantially since initial enrollment. Efficacy in preventing symptomatic, laboratory-confirmed COVID-19 in persons aged ≥16 years without evidence of previous SARS-CoV-2 infection was 91.1% (Table 1). No hospitalizations were reported for confirmed COVID-19 in the vaccinated group and 31 confirmed COVID-19–associated hospitalizations in the placebo group, yielding an estimated vaccine efficacy of 100% against COVID-19 hospitalization. One death attributed to COVID-19 occurred in the vaccinated group and six in the placebo group, resulting in a vaccine efficacy of 83.3% against death attributed to COVID-19. The clinical trial did not routinely collect specimens to test for asymptomatic SARS-CoV-2 infection. Observational data were available for all beneficial outcomes assessed. The pooled vaccine effectiveness estimates from observational studies were 92.4% for prevention of symptomatic, laboratory-confirmed COVID-19; 94.3% against COVID-19–related hospitalization; 96.1% against death attributed to COVID-19; and 89.3% against asymptomatic SARS-CoV-2 infection. Although some studies covered recent periods, most follow-up time occurred before widespread circulation of the SARS-CoV-2 B.1.617.2 (Delta) variant.

**TABLE 1:** Potential benefits of Pfizer-BioNTech COVID-19 vaccination: Grading of Recommendations, Assessment, Development and Evaluation — United States, September 2021

Potential benefit	Clinical trial evidence		Observational evidence		GRADE evidence type^†^
	No. of studies	Vaccine efficacy (95% CI)	No. of studies	Pooled vaccine effectiveness* (95% CI)	
Prevention of symptomatic laboratory-confirmed COVID-19^§^	1	91.1 (88.8 to 93.1)	8	92.4 (87.5 to 95.3)	1
Prevention of COVID-19–associated hospitalization^§^	1	100 (87.6 to 100)	8	94.3 (87.9 to 97.3)	2
Prevention of COVID-19–associated death	1	83.3 (−38.6 to 98.0)	4	96.1 (91.5 to 98.2)	2
Prevention of asymptomatic SARS-CoV-2 infection	0	No data	2	89.3 (88.4 to 90.1)	4

**Abbreviations:** CI = confidence interval; GRADE = Grading of Recommendations, Assessment, Development and Evaluation.

* Vaccine effectiveness estimates were pooled for the purposes of GRADE review. https://www.cdc.gov/vaccines/acip/recs/grade/covid-19-pfizer-biontech-vaccine.html

^†^ GRADE evidence types: 1 = high certainty, 2 = moderate certainty, 3 = low certainty, 4 = very low certainty.

^§^ Considered a critical outcome in GRADE. https://www.cdc.gov/vaccines/acip/recs/grade/about-grade.html

Severe local and systemic adverse reactions (i.e., reactogenicity) occurring in the 7 days after vaccination (grade 3 or higher, defined as interfering with daily activity) were more likely to be reported among vaccine recipients (10.7%) than placebo recipients (2.3%) (relative risk = 4.7) (Table 2). Among vaccine recipients, the most common grade 3 symptoms were fatigue, headache, chills, muscle pain, fever, and injection site pain. Overall, reactions consistent with grade 3 or higher were more likely after the second dose than after the first dose. The frequency of serious adverse events^††^ was 1.2% among both vaccine recipients and placebo recipients. Data on serious adverse events from VAERS and VSD demonstrated anaphylaxis and myocarditis/myopericarditis,^§§^ which were rare but occurred after vaccination. VAERS and VSD estimated 4.7 and 5.0 cases of anaphylaxis per million doses of Pfizer-BioNTech COVID-19 vaccine administered, respectively. Myocarditis and myopericarditis were more common among vaccine recipients who were younger, were male, and received the second dose of vaccine. The VSD evaluation included chart-reviewed confirmed myocarditis cases among persons aged 18–39 years after dose 2; the rates of myocarditis were 368 per 1 million person-years (9 of 24,432) in the 0–7-day risk interval compared with 48 per 1 million person-years (3 of 62,481) in the 22–42-day comparison interval among vaccinated persons (adjusted^¶¶^ rate ratio = 9.1). Although VAERS data are subject to the limitations inherent in a passive surveillance system,*** the elevated number of observed versus expected myocarditis cases in the 7-day interval after receipt of dose 2 of Pfizer-BioNTech vaccine is consistent with the findings from VSD (Table 2).

**TABLE 2:** Potential harms of Pfizer-BioNTech COVID-19 vaccination: Grading of Recommendations, Assessment, Development and Evaluation — United States, September 2021

Potential harms	Clinical trial evidence		Observational evidence		GRADE evidence type*
	No. of studies	RR (95% CI)	No. of studies	Cases per million doses; RR (95% CI); observed versus expected cases	
**Reactogenicity**	2	4.7 (3.8–5.7)	0	No data	1
**Serious adverse events^†^**	2	1.0 (0.8–1.2)	0	No data^§^	2
** Anaphylaxis**					
Persons aged ≥16 yrs, VAERS	NA	NA	1	4.7 cases per million doses^¶^	3
Persons aged ≥12 yrs, VSD	NA	NA	1	5.0 cases per million doses**	
** Myocarditis**					
Males and females aged 18–39 yrs, VSD	NA	NA	1	RR = 9.1 (95% CI = 2.1–48.6)^††^	3
Males aged 16–17 yrs, VAERS	NA	NA	1	120 cases observed versus 0–3 expected^§§^	
Females aged 16–17 yrs, VAERS	NA	NA	1	15 cases observed versus 2 expected^§§^	
Males aged 30–39 yrs, VAERS	NA	NA	1	40 cases observed versus 1–11 expected^¶¶^	
Females aged 30–39 yrs, VAERS	NA	NA	1	7 cases observed versus 1–13 expected^¶¶^	

**Abbreviations:** CI = confidence interval; GRADE = Grading of Recommendations, Assessment, Development and Evaluation; NA = not applicable; RR = relative risk; VAERS = Vaccine Adverse Event Reporting System; VSD = Vaccine Safety Datalink.

* GRADE evidence types: 1 = high certainty, 2 = moderate certainty, 3 = low certainty, 4 = very low certainty.

^†^ Considered a critical outcome in GRADE. https://www.cdc.gov/vaccines/acip/recs/grade/about-grade.html

^§^ Observational evidence did not include an aggregate measure of serious adverse events. Data on specific serious adverse events identified through post-authorization safety surveillance were reviewed. Increased risk for myocarditis and anaphylaxis were observed in VAERS and VSD.

^¶^ Based on VAERS passively reported cases, in persons aged ≥16 years, occurring in a 0–1-day risk interval after vaccination.

** Based on VSD chart reviewed cases of anaphylaxis, in persons aged ≥12 years, occurring in a 0–1-day risk interval after vaccination (RR = 5.0; 95% CI = 3.5–6.9).

^††^ Based on VSD chart-reviewed cases of myocarditis occurring among persons aged 18–39 years after dose 2, occurring in a 7-day risk interval after vaccination (368 per million person-years) versus a 22–42 day comparison interval in vaccinated persons (48 per million person-years). Adjusted for VSD site, 5-year age group, sex, race/ethnicity, and calendar date.

^§§^ Based on VAERS chart-reviewed cases of myocarditis among males and females aged 16–17 years compared with baseline expected cases in the absence of vaccination.

^¶¶^ Based on VAERS preliminary reports of myocarditis among males and females aged 30–39 years compared with baseline expected cases in the absence of vaccination.

From the GRADE evidence assessment, the level of certainty for the benefits of Pfizer-BioNTech COVID-19 vaccination among persons aged ≥16 years was type 1 (high certainty) for the prevention of symptomatic COVID-19, type 2 (moderate certainty) for prevention of hospitalization and death attributed to COVID-19, and type 4 (very low certainty) for prevention of asymptomatic SARS-CoV-2 infection (Table 1). Regarding potential harms after vaccination, evidence was type 2 for serious adverse events and type 1 for reactogenicity (Table 2). Since the interim recommendations for Pfizer-BioNTech COVID-19 vaccine were issued in December 2020 (*1*), data have become available on all outcomes of interest, and the level of certainty in the estimates of the vaccine benefits has increased for prevention of hospitalization and death. The GRADE evidence profile is available at https://www.cdc.gov/vaccines/acip/recs/grade/covid-19-pfizer-biontech-vaccine.html.

Data reviewed within the EtR framework support the use of the Pfizer-BioNTech COVID-19 vaccine. The COVID-19 Vaccines Work Group concluded that COVID-19 remains an important public health problem and that the desirable effects of disease prevention via vaccination with Pfizer-BioNTech COVID-19 vaccine in persons aged ≥16 years are large and outweigh the potential harms. The Work Group determined that the vaccine is acceptable to vaccine providers and that implementation of vaccination is feasible. Moreover, full FDA approval might increase acceptability of the vaccine among unvaccinated persons. The Work Group also acknowledged that vaccine-eligible persons aged ≥16 years probably considered the desirable effects of vaccination to be favorable compared with the undesirable effects; however, there is likely important variability in vaccine acceptance within this age group, especially among those who are currently unvaccinated. The Work Group had varying opinions regarding the impact that a standard ACIP recommendation for Pfizer-BioNTech COVID-19 vaccine would have on health equity; most felt the impact might vary by subpopulation. The evidence used to guide the EtR is available at https://www.cdc.gov/vaccines/acip/recs/grade/covid-19-pfizer-biontech-etr.html.

In summary, after 8 months of use under an FDA EUA and ACIP interim recommendation, the Pfizer-BioNTech COVID-19 vaccine, Comirnaty, now has full FDA approval and is recommended by ACIP for use in persons aged ≥16 years in the United States. Comirnaty has the same formulation and can be used interchangeably with the Pfizer-BioNTech COVID-19 vaccine used under EUA without presenting any safety or effectiveness concerns. ACIP considered new information beyond what was available at the time of the interim recommendation: an additional 4 months of follow-up of phase II/III clinical trial participants, 26 observational vaccine effectiveness studies involving hundreds of thousands of vaccinated persons, and two postauthorization safety monitoring systems that encompassed millions of vaccinated persons in the United States. The additional information increased certainty that the benefits of Pfizer-BioNTech COVID-19 vaccine outweigh vaccine-associated risks. The Pfizer-BioNTech COVID-19 vaccine continues to have FDA authorization for emergency use and ACIP interim recommendation for use in adolescents aged 12–15 years (*2*), as well as an additional dose in persons aged ≥12 years who are moderately to severely immunocompromised (*7*).

Before vaccination, a Fact Sheet or Vaccine Information Sheet should be provided to recipients and parents or guardians. Providers should counsel Pfizer-BioNTech COVID-19 vaccine recipients and parents or guardians about expected systemic and local reactogenicity. Additional clinical considerations for COVID-19 vaccine administration are available at https://www.cdc.gov/vaccines/covid-19/info-by-manufacturer/pfizer/clinical-considerations.html.

## Reporting of Vaccine Adverse Events

Providers are required to report adverse events (including administration errors, serious adverse events, cases of multisystem inflammatory syndrome, and cases of COVID-19 that result in hospitalization or death) that occur after receipt of any COVID-19 vaccine to VAERS (*8*). Information on how to submit a report to VAERS is available at https://vaers.hhs.gov/index.html or 1-800-822-7967. Any person who administers or receives a COVID-19 vaccine is encouraged to report any clinically significant adverse event, regardless of whether it is clear that a vaccine caused the adverse event. In addition, CDC has developed a voluntary smartphone-based online tool (v-safe) that uses text messaging and online surveys to provide near real-time health check-ins after receipt of a COVID-19 vaccine. Adult vaccine recipients can register in v-safe, and parents or guardians can register their adolescent children in v-safe and complete the health surveys on their behalf. CDC’s v-safe call center follows up on reports to v-safe that include possible medically significant health events to collect additional information for completion of a VAERS report. Information on v-safe is available at https://www.cdc.gov/vsafe.

**Summary**

**What is already known about this topic?**

On August 23, 2021, the Food and Drug Administration granted full approval of the Pfizer-BioNTech COVID-19 vaccine for persons aged ≥16 years.

**What is added by this report?**

On August 30, 2021, after a systematic review of the data, the Advisory Committee on Immunization Practices revised its interim recommendation to a standard recommendation for use of the Pfizer-BioNTech COVID-19 vaccine in persons aged ≥16 years for the prevention of COVID-19.

**What are the implications for public health practice?**

Continued use of the Pfizer-BioNTech COVID-19 vaccine, now fully approved by the FDA in persons aged ≥16 years, is recommended based on increased certainty that its benefits (prevention of asymptomatic infection, COVID-19, and associated hospitalization and death) outweigh vaccine-associated risks.

## Footnotes

*
https://covid.cdc.gov/covid-data-tracker/#vaccinations

†
https://www.cdc.gov/vaccines/acip/recs/grade/downloads/acip-evidence-recs-framework.pdf

§
https://www.cdc.gov/vaccines/acip/recs/grade/about-grade.html

¶ On August 30, 2021, ACIP voted unanimously in favor of the recommendation for use of Pfizer-BioNTech COVID-19 vaccine for persons aged ≥16 years under the FDA Biologics License Application.

**
https://www.cdc.gov/vaccines/acip/meetings/downloads/slides-2021-08-30/07-COVID-Gargano-508.pdf

†† Serious adverse events are defined as any untoward medical occurrence that results in death, is life-threatening, requires inpatient hospitalization or prolongation of existing hospitalization, or results in persistent disability or incapacity; suspected transmission of any infectious agent via a medicinal product; and a medically important event.

§§ Myocarditis is an adverse event defined as inflammation of the heart muscle. If it is accompanied by pericarditis, an inflammation of the thin tissue surrounding the heart (the pericardium), it is referred to as myopericarditis.

¶¶ Rate ratio adjusted for VSD site, 5-year age group, sex, race/ethnicity, and calendar date.

*** Because VAERS is a passive reporting system, limitations include possible bias in reporting, inconsistent data quality, and incomplete information; in addition, VAERS has no direct comparison group. The VAERS system was not designed to assess causality; therefore, VAERS data generally cannot be used to determine whether a causal association between an adverse event and a vaccine exists.
